# The proteomics analysis of extracellular vesicles revealed the possible function of heat shock protein 60 in *Helicobacter pylori* infection

**DOI:** 10.1186/s12935-023-03131-1

**Published:** 2023-11-16

**Authors:** Yujie Li, Hui Cao, Dewen Qiu, Nan Wang, Yan Wang, Tingting Wen, Jianjun Wang, Hong Zhu

**Affiliations:** 1 Department of Clinical Laboratory, Kunshan Hospital Affiliated to Jiangsu University, Suzhou, 215300 Jiangsu People’s Republic of China; 2 Department of Food and Nutrition Safety, Jiangsu Provincial Center for Disease Control and Prevention, Nanjing, 210009 Jiangsu People’s Republic of China; 3 Department of Clinical Laboratory, Jiangxi Maternal and Child Health Hospital Maternal and Child Heath Hospital of Nanchang College, Nanchang, 215300 People’s Republic of China; 4 The School of Medicine, Jiangsu University, Zhenjiang, 212013 Jiangsu People’s Republic of China; 5 Department of Pharmacy, First People’s Hospital of Kunshan, Suzhou, 215300 Jiangsu People’s Republic of China; 6 Department of Clinical Laboratory, The Affiliated Changzhou No.2 People’s Hospital of Nanjing Medical University, Changzhou, 213000 People’s Republic of China

**Keywords:** *H. pylori*, Extracellular vesicle, Proteomics, HSP60

## Abstract

**Background:**

*Helicobacter pylor*i (*H. pylori*) infection is a major risk factor for gastric diseases, including gastritis and gastric cancer. Heat shock protein 60 (HSP60) is a chaperone protein involved in various cellular processes and has been implicated in the immune response to bacterial infections. Extracellular vesicles (EVs) containing various protein components play important roles in cell communication. In the present study, a systematic proteomic analysis of EVs obtained from *H. pylori* infected cells was performed and the EV-derived HSP60 function was studied.

**Methods:**

EVs were evaluated by nanoparticle tracking analysis, transmission electron microscopy and western blotting. The recognized protein components were quantified by label-free proteomics and subjected to bioinformatics assays. The expression of HSP60 in EVs, host cells and gastric cancers infected by *H. pylori* was determined by western blotting and immunohistochemical, respectively. In addition, the apoptotic regulation mechanisms of HSP60 in *H. pylori* infection were analyzed by western blotting and flow cytometry.

**Results:**

A total of 120 important differential proteins were identified in the EVs from *H. pylori*-infected cells and subjected to Gene Ontology analysis. Among them, CD63, HSP-70 and TSG101 were verified via western blotting. Moreover, HSP60 expression was significantly increased in the EVs from *H. pylori*-infected GES-1 cells. *H. pylori* infection promoted an abnormal increase in HSP60 expression in GES-1 cells, AGS cells, gastric mucosa and gastric cancer. In addition, knockdown of HSP60 suppressed the apoptosis of infected cells and the expression of Bcl2, and promoted the upregulation of Bax.

**Conclusion:**

This study provides a comprehensive proteomic profile of EVs from *H. pylori*-infected cells, shedding light on the potential role of HSP60 in *H. pylori* infection. The findings underscore the significance of EV-derived HSP60 in the pathophysiology of *H. pylori*-associated diseases.

**Supplementary Information:**

The online version contains supplementary material available at 10.1186/s12935-023-03131-1.

## Background

*Helicobacter pylori (H. pylori)*, a main Gram-negative bacterium, asymptomatically colonizes more than 50% of the global population and is associated with numerous gastrointestinal diseases, such as gastritis, gastric ulcer and gastric cancer (GC) [1]. The identification of the involvement of *H. pylori* infection causes gastrointestinal diseases was a landmark [2, 3]. *H. pylori*, as an inducer of GC, is considered a group 1 carcinogen by the World Health Organization [4]. Although 10% of peptic ulcer disease is associated with *H. pylori* infection, 1–3% of those infected patients will develop to GC, and survival is < 5 years [5]. Another 0.1% of patients will suffer mucosa-related lymphoid tissue lymphoma [6]. The fact that *H. pylori* infection causes gastritis and GC has been recognized worldwide, but the specific molecular mechanism remains unclear.

Extracellular vesicles (EVs) are small, membrane-bound vesicles that are released by all cells in the body [7–9]. EVs can contain a variety of biomolecules, including proteins, nucleic acids, and lipids [11–13]. EVs play a role in cell communication and can also be used by pathogens to deliver virulence factors to host cells [10]. Studies have shown that EVs from *H. pylori*-infected gastric epithelial cells can contain a variety of virulence factors, including the cytotoxin-associated gene A (CagA) protein [14]. CagA is a major virulence factor of *H. pylori* and is thought to play a role in the development of gastric cancer [15]. EVs from *H. pylori*-infected can also interact with host cells and modulate their immune response. For example, *H. pylori* EVs can induce the production of inflammatory cytokines and suppress the activity of T cells [16, 17]. This can lead to the development of chronic inflammation in the stomach, which is a risk factor for gastric cancer [16, 17]. In addition to their role in the pathogenesis of *H. pylori* infection, EVs from *H. pylori*-infected cells may also be used as diagnostic and therapeutic targets. For example, *H. pylori* EVs can be detected in the blood of patients with *H. pylori* infection, which could be used to develop a non-invasive diagnostic test for *H. pylori* infection [16]. Overall, research on the relationship between EVs from *H. pylori*-infected cells and gastric diseases is still in its early stages, and in particular, there is a lack of systematic studies on the profiling of these EVs and the function of EV components.

Heat shock protein 60 (HSP60) is a highly conserved protein that plays a vital role in protein folding and assembly. It is also involved in other cellular processes, such as stress response, immunity, and apoptosis [18]. There is growing evidence that human HSP60 plays a role in the pathogenesis of a variety of diseases, including cancer, autoimmune diseases, and infectious diseases [19–21]. HSP60 has been shown to promote tumor growth and progression, suppress the anti-tumor immune response, and promote the development of gastric cancer stem cells [22]. However, the specific mechanism underlying these activities of HSP60 remains to be further explored.

Proteomics offers complementary information to genomics and transcriptomics, and is essential for the understanding of complex biochemical processes at the molecular level [23]. Mass spectrometry (MS) has emerged as the most important and popular tool to identify, characterize and quantify proteins and their post-translational modifications (PTMs) with high throughput and on a large scale [24]. The present study screened by proteomics and bioinformatics analysis certain differentially expressed proteins in *H. pylori-*infected gastric epithelial cells. These candidate differential proteins in EVs were further confirmed. Furthermore, the expression of heat shock protein (HSP) 60 in clinical samples was evaluated, and its functions in the *H. pylori-*infection process were investigated.

## Methods

### Ethics statement

Gastric mucosa samples of 30 normal individual and 30 patients with *H. pylori* infection were obtained from the Kunshan First People's Hospital from February to December 2021. The samples were then clipped by endoscopy and verified by urease test. All samples were kept at − 80 °C for further experiments. GC tissue microarray (66 paracancerous and 68 complete GC tissues, including 26 *H. pylori*-infected GC tissues; cat. no. HStmA180su30-T-113 RG-K-16-C13) was purchased from Shanghai Xinchao Biotechnology Co., Ltd.

Ethics approval was obtained from the institutional Ethics Committee of Kunshan first people's Hospital (Suzhou, China, approval no. 2021-06-014-K01). The experiments were conducted as per the Declaration of Helsinki, and all participants offered written informed consent.

### Cell culture and *H. pylori* infection

GES-1 and AGS cells were purchased from the Cell Bank of Type Culture Collection of Chinese Academy of Sciences (Shanghai, China). GES-1 and AGS cells were cultivated in culture dishes with DMEM supplemented with 10% FBS, 100 U/ml penicillin and 0.1 mg/ml streptomycin at 37 °C with 5% CO_2_. Both GES-1 and AGS cells were used in the current study for *H. pylori* infection and EV production.

*H*. *pylori* (NCTC 11637 strain) was donated by Marshall International Digestive Disease Diagnosis and Treatment Center of Shanghai Oriental Hospital. *H*. *pylori* was cultured on Columbia blood agar plates and incubate in a microaerophilic chamber at 37 °C for 3–4 days. Infection was conducted at a multiplicity of cells: *H*. *pylori* = 1:50 for 24 h, and cleaned with PBS three times.

### Isolation of EVs

Cell culture supernatants from either uninfected or *H*. *pylori*-infected GES-1 cells for 48 h were collected. EVs from culture supernatants were centrifuged at 300×*g* for 10 min, 3000×*g* for 15 min, and 10,000×*g* for 60 min to remove cells, cell debris, bacteria and microvesicles respectively. Then pellets therefrom were resuspended with 200 μl of 9% sucrose containing protease inhibitors and centrifuged at 100,000*g* for 2 h. Each step was followed by two washes with PBS at 4 °C. EVs were resuspended with 50 μl of 9% sucrose containing protease inhibitors and stored at − 80 °C before further experiments.

### Transmission electron microscopy (TEM)

An EVs diluted solution (10 μl) was put onto the copper wire at 25 °C for 5 min, and washed three times with ultrapure water in triple. After air drying, 10 μl of a phosphotungstic acid solution was added to the copper mesh coloring for 2 min for coloring, and then air dried at ambient temperature. The morphology of the EVs was observed under a Hitachi H-600 transmission electron microscope (Hitachi, Ltd., Tokyo, Japan).

### Nanoparticle size analysis

EVs were diluted to 10^6^ EVs/ml with 100 μl PBS. Upon homogenization, EVs (10 μl) were diluted to 1 ml with ultrapure water, and placed into a NanoSight NS300 nanoparticle size analyzer for measurement of particle size distribution and concentration following standard procedures at 4 °C. The laser beam passed through EVs outside the sample room; achieved particle visualization through a microscope equipped with a camera; captured the Brownian motion of EVs; and used software to calculate the concentration and hydrodynamic diameter according to their motion. The diameter and concentration of the EVs were obtained through NanoSight Nanoparticle tracking analysis (NTA) 3.2 software analysis.

### Protein isolation and digestion

EVs samples were isolated with an SDT buffer (4% SDS, 100 mM Tris–HCl and 1 mM DTT, pH 7.6). Protein levels were detected with a BCA assay kit (Bio-Rad Laboratories, Inc.). Next, the EV proteins were digested with trypsin as per the filter-aided sample preparation (Matthias Mann). The obtained peptides were desalted by using Empore SPE Cartridges C18 [standard density, bed internal diameter (I.D.), 7 mm; volume, 3 ml; Sigma-Aldrich; Merck KGaA], vacuum-centrifuged and reconstructed in 40 µl 0.1% (v/v) formic acid. Proteins (200 μg) were placed into 30 μl another SDT buffer (4% SDS, 100 mM DTT and 150 mM Tris–HCl pH 8.0). The detergent, DTT and other low-molecular-weight chemicals were ultrafiltered numerous times with a UA buffer (8 M urea and 150 mM Tris–HCl, pH 8.0) (Microcon; 10 kDa). Next, the reduced cysteine residues were blocked with 100 μl iodoacetamide (100 mM in UA buffer) and incubated for 30 min in the dark. The filters were washed first with 100 μl UA buffer three times, and then with 100 μl 25 mM NH_4_HCO_3_ twice. Finally, the protein suspensions were digested with 4 μg trypsin (Promega Corporation) in 40 μl NH_4_HCO_3_ buffer at 37 °C overnight, and the final peptides were collected as a filtrate.

The ultraviolet density at 280 nm of the peptides was detected with an extinction coefficient of 1.1 of a 0.1% (g/l) solution, which was calculated based on the frequency of tryptophan and tyrosine in vertebrate proteins.

### Liquid chromatography with tandem mass spectrometry (LC–MS/MS)

LC–MS/MS was conducted on a Q-Exactive MS meter with EASY nLC for 120 min (Thermo Fisher Scientific, Inc.). The peptides were placed into a PepMap100 reverse trap column (Acclaim, 100 μm × 2 cm, nanoViper C18; Thermo Fisher Scientific, Inc.) connected to a C18 reversed column (Easy, 10-cm long, 75 μm I.D., 3 μm resin) in buffer A (0.1% formic acid), and separated with a linear gradient of buffer B (84% acetonitrile, 0.1% formic acid) at a rate of 300 nl/min controlled on IntelliFlow. MS data in the positive ion mode were acquired using a data-based top10 method to dynamically select the richest precursor ions from 300 to 1800 m/z for hierarchical cellular decomposition (HCD) decomposition. The largest injection time, gain auto-control target and dynamic exclusion duration were 10 ms, 3e6 and 40.0 s, respectively. Survey scanning resolution, HCD resolution and isolation width were 70,000 at m/z 200, 17,500 at m/z 200, and 2 m/z, respectively. The normalized collision energy was 30 eV. The under fill ratio, which was the lowest percentage of the target value to be met at the longest fill time, was 0.1%. The peptide recognition mode was adopted. The raw MS data were combined and searched on MaxQuant 1.5.3.17 for identification and quantification.

### Bioinformatic analysis

HCD was conducted with Cluster 3.0 (http://bonsai.hgc.jp/mdehoon/software/cluster/software.htm). Tree diagrams, heat maps and volcano plot were adopted. The subcellular positioning of EV proteins was predicted with a Cell Ontology system (http://cello.life.nctu.edu.tw/) [25]. With the KEGG database (http://geneontology.org/) [26], the EV differential proteins were blasted to identify their KEGG orthology, and then mapped to the path in KEGG path.

Total quantitative protein was used as the background dataset in the enrichment analysis based on Fisher’s exact test. P < 0.05 was considered to indicate a statistically significant difference. Protein–protein interactions (PPIs) were represented to express the molecular mechanisms and signaling pathways of cell processing. The PPIs of EV proteins were explored with Search Tool for the Retrieval of Interacting Genes/Proteins (http://string.embl.de/) [27], and then was visualized with Cytoscape (https://cytoscape.org/) [28].

### Cell transfection

HSP60 small interfering RNA (siRNA) and scrambled siRNA (50 nM; GenePharma Co., Ltd.) were transfected into GES-1 cells cultured in 6-well plates at 75% confluence using Lipofectamine 3000® reagent (Invitrogen; Thermo Fisher Scientific, Inc., Waltham, MA, USA) according to the manufacturer’s protocols at 37 °C for 24 h. The HSP60 siRNA sense sequence was 5′-GACGAUGCCAUGCUCUUAAT-3′, and the antisense sequence was 5′-UUAAGAGCAUGGCAUCGUCTT-3′, while the scrambled siRNA sequence was 5′-UUCUCCGAACGUGUCACGUTTACGUGACACGUUCGGAGAATT-3′.

### Apoptosis assay

Four treated cells groups (untreated GES-1 cell control, scrambled siRNA, siRNA and siRNA + Hp) were collected and washed twice with PBS. Cells were re-suspended in 100 μl binding buffer, and then incubated for 15 min with Annexin-V and PI (each 5 μl) (BD Biosciences Company, USA) in the dark at 37 °C. Subsequently, cells in 500 μl binding buffer were detected on a FACS Canto II flow cytometer (BD Biosciences Company). The results of apoptosis were analyzed by FlowJo 7.6 software (Tree Star, Inc., Ashland, OR, USA) and GraphpadPrism5 software (GraphPad Software; Dotmatics).

### Western blotting

Total EV or cellular proteins were separated and lysed using a RIPA peptide lysis buffer (Beyotime Institute of Biotechnology, Haimen, China). Proteins (30 μg) were loaded onto 10% SDS-PAGE gels, electrophoresed, and transferred to PVDF membranes (MilliporeSigma, Burlington, MA, USA). Next, the membranes were incubated with primary antibodies against CD63 (1:10,000), HSP70 (1:10,000), ERK1/2 (1:1400), HSP60 (1:10,000), Bcl2 (1:6000), Bax (1:8000), GAPDH (1:200,000), and TSG101 (1:20,000) (all from ProteinTech Group Inc., Chicago, USA) at 4 °C overnight. Next, the membranes were washed three times with TBS-Tween 20 under shaking, incubated with a secondary antibody solution (mouse anti-rabbit IgG-HRP at 1:6,000 dilution) at room temperature under mild shaking for 1.5 h, and re-washed with TBS-T under shaking three times. Protein bands were photographed and examined with a chemiluminescent substrate system (Thermo Fisher Scientific, Inc.). The intensity of the western blot was determined with ImageJ software and analyzed by GraphPad Prism 5 software (GraphPad Software; Dotmatics).

### Multicolor immunohistochemistry (IHC)

For multicolor staining, GC tissue arrays with 66 paracancerous and 68 complete GC tissues, including 26* H. pylori-infected* GC tissues (Shanghai Xinchao Biological Technology Co., Ltd.) were processed for IHC according to the instructions of the manufacturer of the Opal 2-Color Anti-Rabbit Automation IHC Kit (cat. no. NEL830001KT; Akoya Biosciences). Slides were incubated at 4 °C overnight with primary anti-HSP60 (Signaling Technology, Inc.) antibody. Next, the array was incubated with the secondary antibody solution for 50 min at room temperature under light agitation, followed by three washed with PBS, and imaged using a digital microscope camera (Nikon Corporation).

### Statistical analysis

Data are expressed as the mean ± standard error. Statistical analysis was performed with GraphPad Prism 5 (GraphPad Software Inc., San Diego, CA, USA). Groups were compared via one-way ANOVA or paired Student's *t* test. P < 0.05 was considered to indicate a statistically significant difference.

## Results

### Biological features of EVs from *H. pylori*-infected cells

**Fig. 1:**
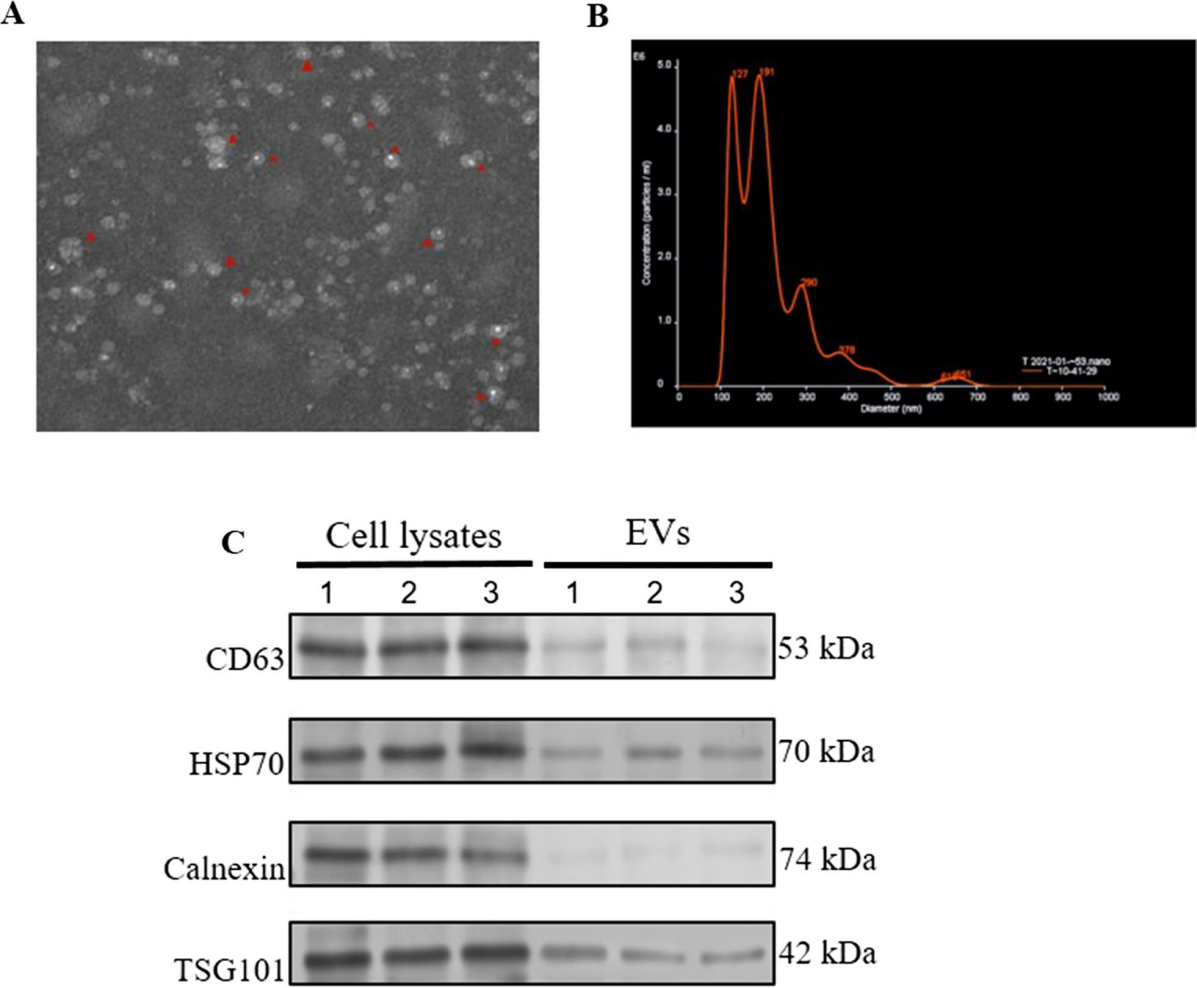
The biological properties of EVs purified from *H. pylori* infected GES-1 cells. **A** The morphological character of EVs under the transmission electron microscopy (magnification of × 200). **B** The size distribution of EVs were determined with NTA. **C** The purity of EVs was determined by western blotting

EVs were first purified from *H. pylori* infected or uninfected GES-1 cells, and then the morphology, size and purity of EVs were determined by transmission election microscopy, nanoparticle tracking analysis and western blot, respectively. As shown in Fig. 1A, EVs were in the typical cup-like vesicles under TEM. Nanoparticle tracking analysis (NTA) showed that the size of the majority of particles was between 30 and 150 nm, and the concentration of particles reached to 5.63 × 10^9^ particles/ml (Fig. 1B and Additional file 1: Fig. S1A-B). In addition, the purity of the EVs were assessed by measuring the presence of 3 positive markers (CD63, HSP70 and TSG101) and 1 negative marker (Calnexin). Our data in Fig. 1F showed that purified EVs had high levels of all 3 positive markers but very low level of the negative marker Calnexin, indicating that the purified EVs were in good purity. Taken together, our data here indicates that purified EVs from *H. pylori* infected or uninfected cells had expected morphology, size, and purity.

### Proteomics analysis of EVs

**Fig. 2:**
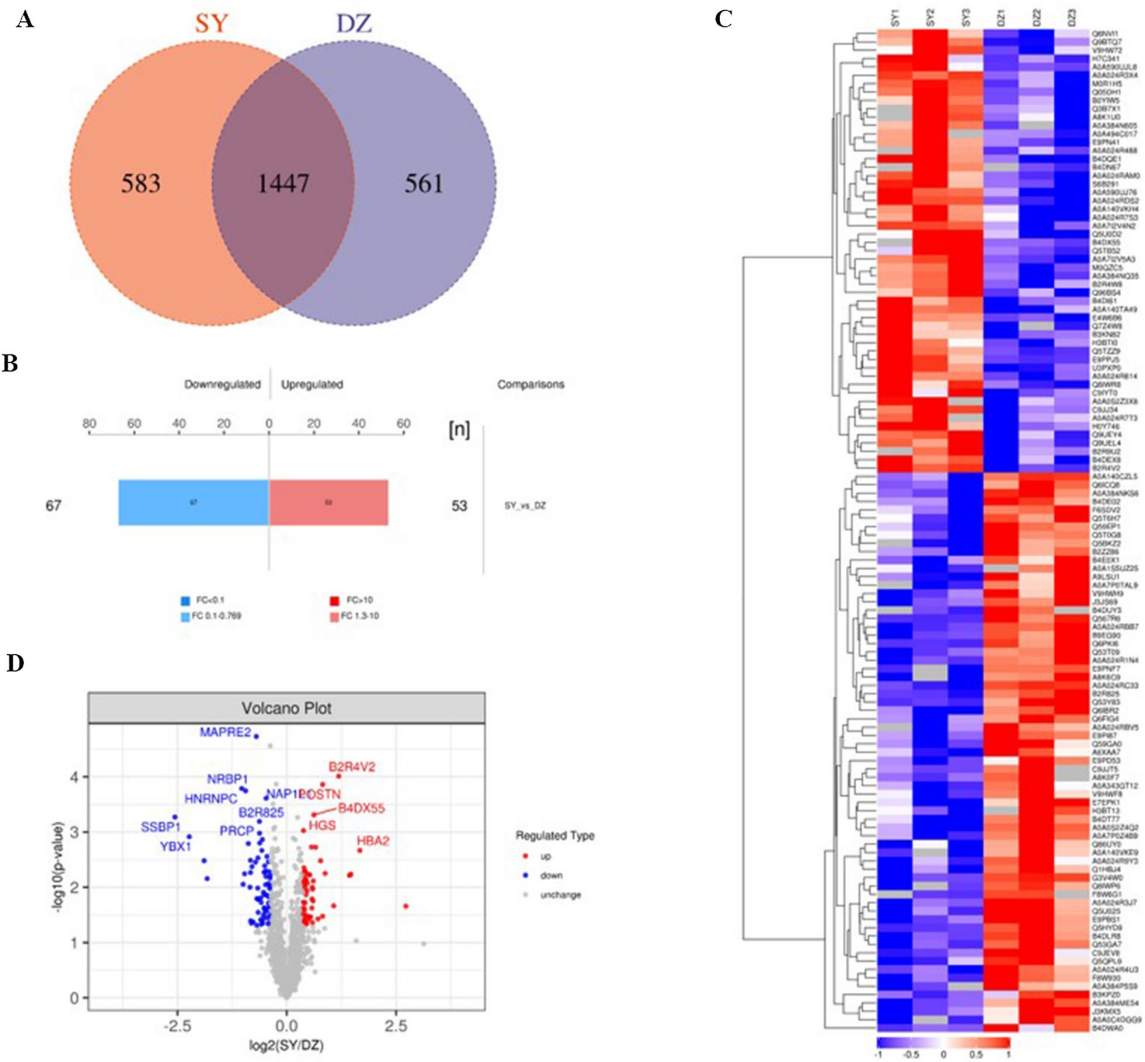
EV differential proteins were analyzed by bioinformatics. **A** The venn diagram illustrated 1447 overlapping proteins in GES-1 infected with and without *H. pylori* (SY vs. DZ). **B** 53 up-regulated and 67 down-regulated EV proteins in two groups were indicated by the histogram of quantitative difference. **C** The heat map displayed the expression intensity of EV differential proteins between two groups (SY vs. DZ). **D** The volcano diagram represented 53 up-regulated and 67 down-regulated EV proteins in two groups and some EV proteins were marked with the unique and stable entry identifier of protein. *Note* DZ: Hp uninfected group, SY: Hp infected group. Red: high expression, blue: low expression

To evaluate the EV protein profiles of *H. pylori*-infected GES-1 cells, total EV proteins were quantified via label-free quantitative proteomics analysis. The results of MS/MS analysis revealed that there were 1447 common EV proteins in the two groups investigated (SY: *H. pylori*-infected GES-1 cells vs. DZ: *H. pylori*-uninfected GES-1 cells), as indicated in Venn diagram show in Fig. 1A. Bioinformatics analysis of the proteomics data indicated that there were 120 EV differential proteins including 53 up-regulated proteins and 67 down-regulated proteins were cluster analysis with 1.5 fold change (Fig. 2B). The heat map and volcano plot results represented the expression levels of 120 EV differential proteins sufficiently (Fig. 2C, D), with red indicating high expression and blue indicating low expression. The 13 upregulated and 14 downregulated proteins of interest in are summarized in Table 1.

**Table 1:** List of 27 EV differential proteins with significant interesting from *H. pylori* infected GES-1 cells

Number	Protein	Protein name	Gene	Proteins	Peptides	Unique peptides	Coverage	Mol/weight	Expression pattern
1	E9PPJ5	Midkine (Fragment)	MDK	2	5	5	39.7	14.374	Up
2	A0A0S2Z3X8	Rab GDP dissociation inhibitor (Fragment)	GDI1	6	13	7	43	50.582	Up
3	Q5TZZ9	Annexin	ANXA1	29	21	21	64.2	38.714	Up
4	A0A590UJ76	Deleted in malignant brain tumors 1 protein	DMBT1	5	3	3	6	260.63	Up
5	Q9UEY4	Anion exchange protein	SLC4A2	16	16	16	23.5	135.58	Up
**6**	**A0A024R3X4**	**60 kDa chaperonin (Fragment)**	**HSPD1**	**24**	**19**	**19**	**46.8**	**61.054**	Up
7	E9PN41	Tetraspanin-4 (Fragment)	TSPAN4	7	4	4	47.7	15.195	Up
8	B4DN67	Kin of IRRE-like protein 1	KIRREL1	3	7	7	16.3	72.721	Up
9	A0A024R7S3	Clathrin light chain	CLTB	3	6	6	26.1	23.181	Up
10	H3BTI0	Cysteine-rich secretory protein LCCL domain-containing 2	CRISPLD2	4	7	7	21.6	55.79	Up
11	Q6NVI1	MARCKS protein (Fragment)	MARCKS	2	8	8	83.4	14.859	Up
12	B4DEX8	S-adenosylmethionine synthase	MAT2A	5	9	7	37.6	39.71	Up
13	A0A494C017	Myeloid-associated differentiation marker (Fragment)	MYADM	7	1	1	18.9	13.153	Up
14	F6SDV2	Tubulointerstitial nephritis antigen-like	TINAGL1	1	11	3	56.4	40.867	Down
15	Q86UY0	TXNDC5 protein	TXNDC5	4	8	8	33.1	40.369	Down
16	Q6FIG4	RAB1B protein	RAB1B	2	8	0	52.7	22.198	Down
17	E9PNF7	Lysosomal Pro-X carboxypeptidase	PRCP	11	5	5	20.8	49.902	Down
18	A0A024RC33	Microtubule-associated protein RP/EB family member 2	MAPRE2	3	9	2	36.7	37.031	Down
19	Q1HBJ4	Mitogen-activated protein kinase	MAPK1	9	8	8	27.2	41.389	Down
20	A0A1S5UZ25	Mediator of ErbB2-driven cell motility 1	Memo1	2	5	1	29.2	30.051	Down
21	Q53Y83	Alpha-galactosidase	GLA	12	8	8	24.9	48.766	Down
22	A8K0F7	Vitamin-K-epoxide reductase (warfarin-sensitive)		2	2	2	11.9	19.775	Down
23	F8W6G1	Nuclear receptor-binding protein	NRBP1	3	5	5	13.8	60.829	Down
24	Q6PKI6	YBX1 protein (Fragment)	YBX1	6	11	3	62	29.374	Down
25	F6SDV2	Tubulointerstitial nephritis antigen-like	TINAGL1	1	11	3	56.4	40.867	Down
26	Q86UY0	TXNDC5 protein	TXNDC5	4	8	8	33.1	40.369	Down
27	Q6FIG4	RAB1B protein	RAB1B	2	8	0	52.7	22.198	Down

### Bioinformatics analyses of EVs derived from *H. pylori*-infected cells

**Fig. 3:**
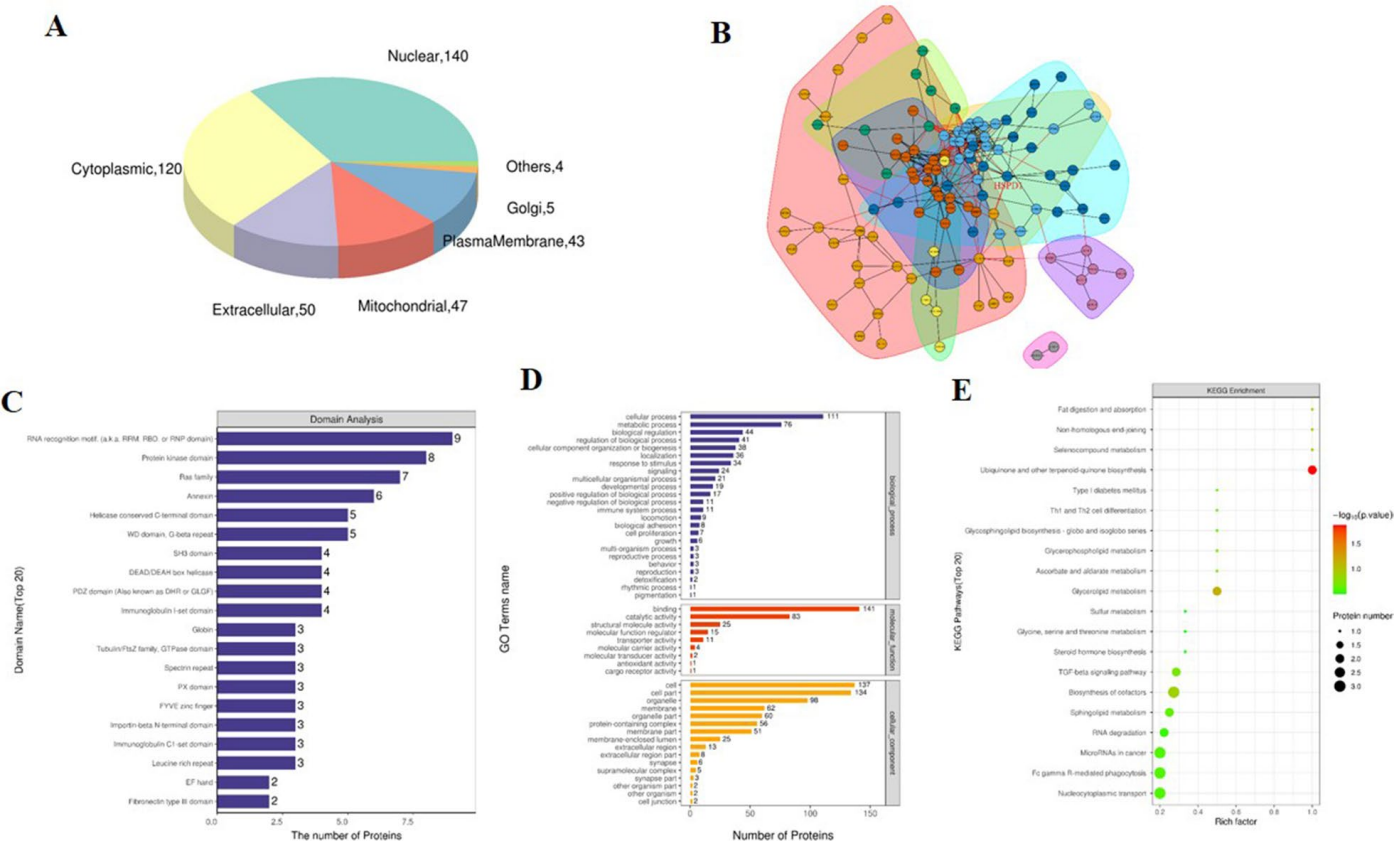
Bioinformatics analysis of EV differential proteins. **A** Subcellular localization pie chart of EV differential proteins in two groups (SY vs. DZ). **B** The interaction network of EV differential proteins. **C** Domain analysis revealed that EV differential proteins involved in many intracellular domains. **D** GO enrichment map including BP, MF and CC showed that EV differential proteins involved in various biological functions, molecular functions and cell structures. **E** Bubble diagram of KEGG pathway enrichment indicated EV differential proteins participated in various intracellular metabolic responses. *Note* DZ: Hp uninfected group, SY: Hp infected group

Bioinformatics analysis of protein function showed that subcellular positioning of EV differential proteins was enriched in the nuclear (140), cytoplasmic (120), extracellular (50), mitochondrial (47) and plasma membrane (43) and other (9) (Fig. 3A). The interaction relationship of EV differential proteins was represented in a network diagram (Fig. 3B), and proteins with the same or similar functions are divided into different clusters with different colors. The interaction network of EV differential proteins showed a complex interaction between EV differential proteins, with the nodes of the HSP60 protein in the network are marked and the proteins interacting with HSP60 is clearly displayed in the diagram.

Domain analysis suggested that EV differential proteins were associated with RNA binding protein domain, protein kinase domain, Ras family and annexin etc. (Fig. 3C**)**. The Gene Ontology (GO) analysis divided the results into biological processes (BP), cellular components (CC) and molecular functions (MF) (Fig. 3D). The results of GO analysis showed that EV differential protein participate in cellular process, metabolic process and biological regulation etc. (BP); binding, catalytic activity and structural molecular activity (MF); and cell, cell part and organelle etc. (CC). The KEGG pathway analysis demonstrated the EV proteins were enriched in ubiquinone and the terpenoid-quinone biosynthesis, glycerolipid metabolism, biosynthesis of cofactors and miRNAs in cancer (Fig. 3E). Bioinformatics analysis revealed that these identified EV proteins were associated with the physiology and pathophysiology of *H. pylori*-infected cells.

### Confirmation of HSP60 expression in *H. pylori*-infected cells and gastric mucosa

**Fig. 4:**
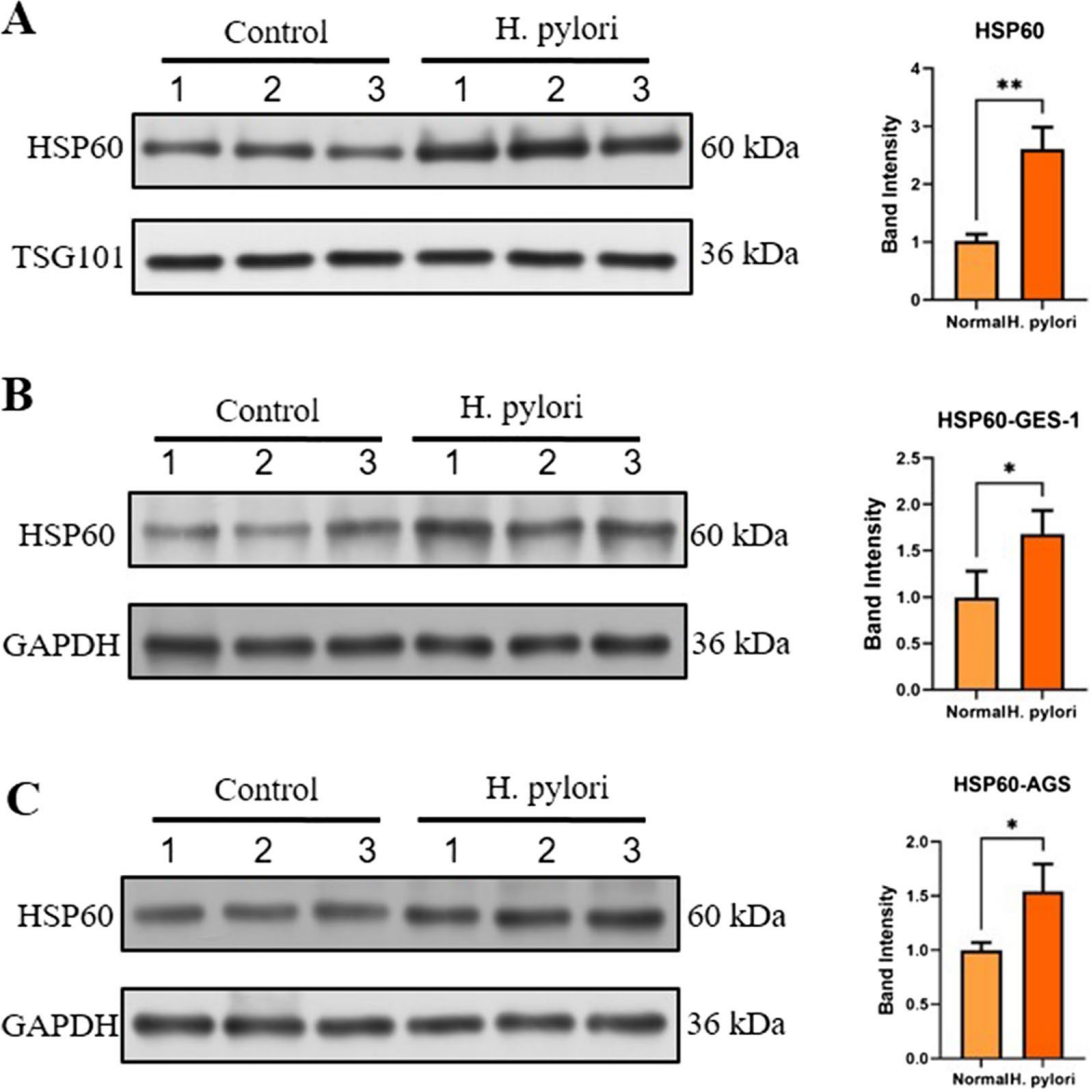
EV differential proteins HSP60 were analyzed in *H. pylori* infected cells and GC tissues. **A**, **B** EV-derived HSP60 in *H. pylori* uninfected group and *H. pylori* infected group (Hp vs control) were detected by western blotting. **B** The relative expression of HSP60 was semiquantified. **C**–**F** The expression levels of HSP60 in *H. pylori* infected (**C**, **D**) GES-1 and (**E**, **F**) AGS cells were determined via western blotting. All western blotting experiments were repeated three times (three panels 1, 2, 3 presents repeated three times). *p < 0.05; **p < 0.01

The upregulation of HSP60 in EVs from *H. pylori*-infected cells was further confirmed by western blot. In consistent with the proteomics data, HSP60 expression was significantly higher in EVs from *H. pylori*-infected cells compared to that from control cells (Fig. 4A, B). HSP60 is active in protecting cell physiology under the stimulation of fever, inflammation and other conditions. We next investigated whether the expression of HSP60 was also increased in *H. pylori*-infected cells. GES-1 and AGS cells infected or noninfected by *H. pylori* was tested for HSP60 expression by western blot, and results showed that HSP60 increased significantly in *H. pylori* infected cells relative to non-infected cells (Fig. 4C–F).

### HSP60 expression in *H. pylori*-infected GC tissues

**Fig. 5:**
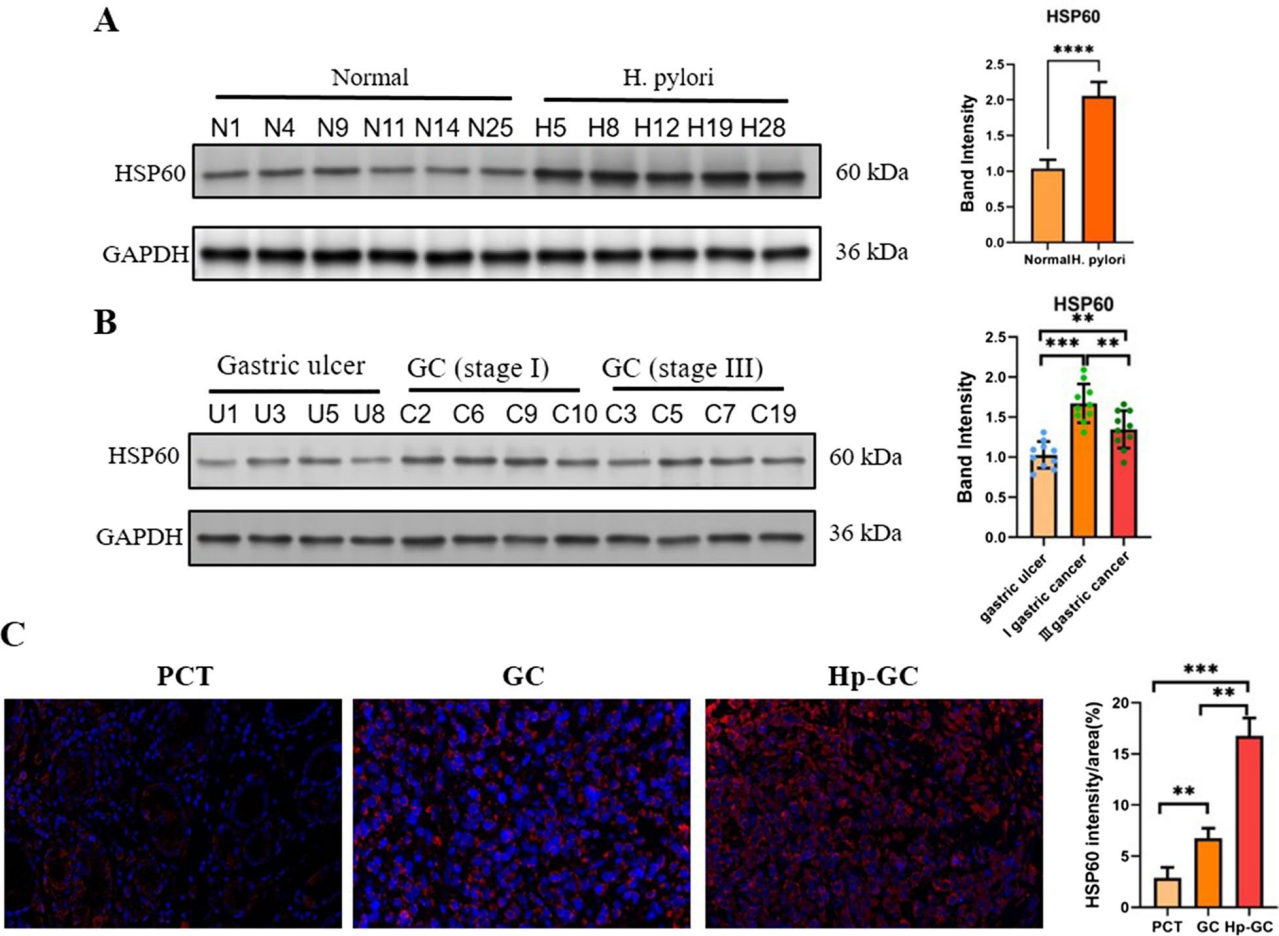
The infection of *H. pylori* regulates the expression of HSP60 in GC. **A** The expression of HSP60 in normal and *H. pylori*-infected mucosal tissues was determined by western blot. The relative expression of HSP60 was semiquantified. **B** The expression of HSP60 in gastric ulcer, and stage I and III GC mucosal tissues was determined by western blot. The relative expression of HSP60 was semiquantified. **C** The expression of HSP60 in PCT, GC and Hp-GC was determined by IHC, and the MFI of HSP60 was also determined. Magnification: 40×. ns, not statistically significant; *p < 0.05; **p < 0.01; ***p < 0.001

To further investigate the association between HSP60 and *H. pylori* infection in vivo, we next tested the expression of *H. pylori* in various gastric tissues. First, the HSP60 level between normal gastric mucosal tissue and *H. pylori*-infected tissue was compared. As shown in Fig. 5A, HSP60 level was around 2-folds higher in *H. pylori*-infected gastric mucosal tissue than in normal tissue. Next, we also checked whether the level HSP60 expression was gastric cancer (GC) stage related. GC stage I and III samples together with gastric ulcer samples as control were collected and the expression of HSP60 was determined by western blot. Our data showed that HSP60 expression was elevated in GC compared to gastric ulcer, but stage III GC did no show further increase in HSP60 expression than stage I GC (Fig. 5B and Additional file 1: Fig. S2). Third, HSP60 expression in paracancerous tissues (PCT), *H. pylori*-negative GC and *H. pylori*-positive GC was also compared. As shown in Fig. 5C, the HSP60 level was significantly upregulated in GC compared to PCT samples, while *H. pylori*-positive GC showed a further drastic increase comparing to *H. pylori*-negative GC.

### HSP60 regulates cell apoptosis

**Fig. 6:**
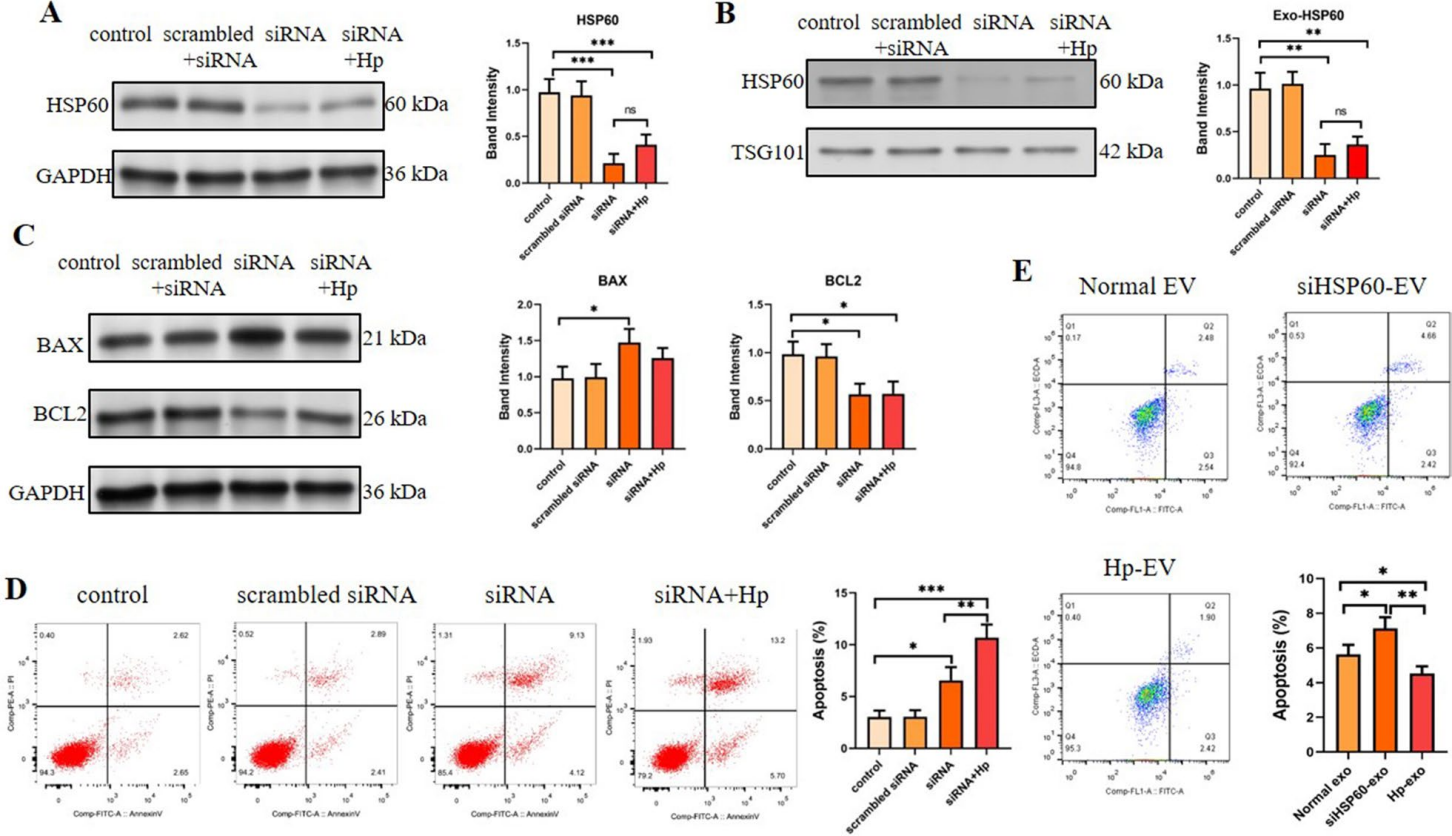
The apoptosis of GES-1 cells regulated by HSP60. **A**–**C** GES-1 cells were first treated with or without HSP60-siRNA, and then either mock-infected or infected with *H. pylori*. Following infection, cells were harvested and the expression of **A** HSP60 in cells, **B** HSP60 and TSG101 in EVs, **C** BAX and BCL2 and **D** apoptosis were measured by western blot and flow cytometry, respectively. **E** GES-1 cells were either untreated, or treated with HSP60-siRNA or infected with *H. pylori*, and then EVs were isolated and their impact on the cell viability of GES-1 cells were determined by flow cytometry. *p < 0.05; **p < 0.01; ***p < 0.001

To explore the function of HSP60 protein in *H. pylori* infection, HSP60 in GES-1 cells was knocked down with siRNA, and cell apoptosis and apoptosis-related proteins BCL1 and Bax in HSP60 knockdown cells were analyzed by flow cytometry and western blotting. The results indicated that the expression of HSP60 was knocked down by siRNA successfully, which resulted in EVs with reduced HSP60 loading (Fig. 6A, B). The knockdown of HSP60 inhibited the expression of the anti-apoptotic protein Bcl2 while enhanced the expression of the apoptotic protein Bax (Fig. 6C). In consistent, apoptosis analysis showed that HSP60 knockdown increased the apoptosis of GES-1 cells (Fig. 6D).

To further explore the function of EV-derived HSP60, EVs from normal cells, HSP60-knocked-down cell and *H. pylori*-infected cells were isolated and their impact on cell viability was assessed. As shown in Fig. 6E, compared with the normal EV group, EVs from HSP60 knocked-down cell demonstrated induced more cell apoptosis, while EVs from *H. pylori*-infected cells showed reduced cell apoptosis. This data indicate that EV-derived HSP60 from *H. pylori*-infected cells could reduce cell apoptosis.

## Discussion

Our bioinformatics analysis identified 120 differentially expressed proteins between the EVs isolated from *H. pylori*-infected and uninfected cells. These proteins are involved in a variety of cellular processes, including metabolism, cell signaling, and immune response. Some of the most notable differentially expressed proteins include peptidylprolyl isomerase, annexin and chaperonin, which have all been shown to be overexpressed in a variety of cancers, including gastric cancer [29–32]. The overexpression of these proteins in the *H. pylori*-infected EV samples suggests that they may play a role in the cancer development and progression. Further research is needed to understand the role of these proteins in gastric cancer and to develop new diagnostic and therapeutic strategies targeting these proteins.

The domain analysis in our current study revealed that the majority of differentially regulated proteins in EVs isolated from *H. pylori*-infected cells were involved in RNA recognition. RNA recognition is the process by which proteins bind to RNA molecules. This process is essential for many cellular functions, including gene expression, RNA processing, and protein translation [33]. A number of RNA recognition proteins have been implicated in the development and progression of gastric cancer [34]. Although beyond the scope of our current study, further research to explore the effects of RNA recognition proteins in EVs from *H. pylori*-infected cells would be warranted. In addition, we observed that HSP60 expression was enhanced in GC tissues compared to gastric ulcer tissues. However, stage III GC did not show higher expression of HSP60 than stage I GC. Because we could not obtain GC samples of intestinal and diffuse types, the expression of HSP60 was not determined in these samples. It would be valuable for future studies to check if there is difference of HSP60 expression in these GC types.

Heat shock proteins (HSPs) participate in protein homeostasis under stresses and heat shock amid normal physiology [35, 36]. HSP60, a nuclear-encoded mitochondrial protein associated with co-chaperonin HSP10, facilitates the folding of proteins to promote the degradation of misfolded or denatured proteins, and participates in immune system activation. It also plays chronic inflammatory and pro-survival or pro-apoptotic roles, and enhances cancer metastasis [37–39]. Chmiela et al. [40] found that HSPB of *H. pylori* and human HSP60 share homology and have immunogenicity, which may cause a cross reaction and further damage to the gastric mucosa. EV differential protein interaction network revealed that HSP60 protein was a key node in the PPI network, and participated in the energy metabolism of mitochondria, the regulation of membrane function and purine metabolism. Furthermore, HSP60 protein interacted with other proteins in the interaction network to regulate cell energy metabolism and maintain cell homeostasis.

According to the results of HSP60 protein in EVs identified by MS, HSP60 proteins were confirmed in *H. pylori*-infected gastric epithelial cells, GC cells and gastric mucosa tissues. Chen et al. [41] reported that HSP60 was an advanced biomarker for digestive system cancer, and its abnormal expression may have implications for early diagnosis in the screening of GC. Notably, HSP60 protein expression increased considerably in GES-1 and AGS cells, and in *H. pylori*-infected gastric mucosa tissues. Additionally, *H. pylori* infection could promote the abnormal increase of HSP60 in GC tissues relative to that in *H. pylori* non-infected GC tissues. HSP60 protein was significantly upregulated in EVs, GES-1 and AGS cells, and GC tissues under *H. pylori* infection. Inactivation of HSP60 affected the key functions of mitochondria, and reduced the respiratory capacity and ATP level of cells [42]. In addition, the release of HSP60 may activate the innate immune system, promoting a proinflammatory state, including an increase in TNF-α [43]. According to the literature, and GO and KEGG analyses, it was concluded that *H. pylori* infection may prevent bacterial infection by upregulating the expression of HSP60 to induce the inflammatory response of cells, and regulate the energy metabolism of cells to maintain normal cell homeostasis.

HSP60 in mitochondria can act on apoptosis-related factors and inhibit the activation of mitochondrial apoptosis pathway, and can reduce the production of oxygen free radicals in mitochondria. A low level of HSP60 in the cytoplasm can also inhibit cell apoptosis through interaction with apoptosis-related factors [44, 45]. Sandeep et al. [40, 46] found that knocking down HSP60 decreased IL-8 expression and its release in prostate cancer cell xenograft tumors in SCID mice, indicating that the HSP60 and IL-8 axis promoted apoptosis resistance in cancer. In the present study, after HSP60 was knocked down in GES-1 cells, the anti-apoptotic protein Bcl2 decreased and the apoptotic protein Bax increased significantly in GES-1 cells. Simultaneously, knockdown of HSP60 promoted cell apoptosis indicating that HSP60 could regulate apoptosis during *H. pylori* infection. Previous studies have indicated that HSP60 may regulate cell apoptosis through BCL2 and BAX [47, 48]. However, the detailed mechanism underlying HSP60-EVs on cell apoptosis remains to be further investigated.

The profiles of the EV proteins in *H. pylori*-infected host cells were further investigated, and the roles of significant differential proteins were explored. The differential proteins associated with *H. pylori* infection, such as HSP60, may be involved in infection, inflammatory responses and cancer induced by *H. pylori*. However, the underlying functions and mechanisms of HSP60 protein, which is highly expressed in EVs and cells under *H. pylori* infection, require further studies.

### Supplementary Information

**Additional file 1: Fig. S1**. NTA analysis of purified exosomes from *H. pylori* infected GES-1 cells. (A) The particle diameter and (B) concentration of exosomes were determined with NTA. **Fig. S2.** The infection of *H. pylori* regulates the expression of HSP60 in GC. The expression of HSP60 in gastric ulcer, and stage I and III GC mucosal tissues was determined by western blot.

## Data Availability

The datasets used and analyzed during the current study are available from the corresponding author on reasonable request.
