# Supplementary material for: The proteomics analysis of extracellular vesicles revealed the possible function of heat shock protein 60 in Helicobacter pylori infection
Source: Cancer Cell Int. 2023 Nov 16;23:272. doi: 10.1186/s12935-023-03131-1 (PMC10652618; doi:10.1186/s12935-023-03131-1)
Supplement: Supplementary file 1 — Additional file 1: Fig. S1. NTA analysis of purified exosomes from H. pylori infected GES-1 cells. (A) The particle diameter and (B) concentration of exosomes were determined with NTA. Fig. S2. The infection of H. pylori regulates the expression of HSP60 in GC. The expression of HSP60 in gastric ulcer, and stage I and III GC mucosal tissues was determined by western blot. [file 12935_2023_3131_MOESM1_ESM.docx]

**Supplementary materials**


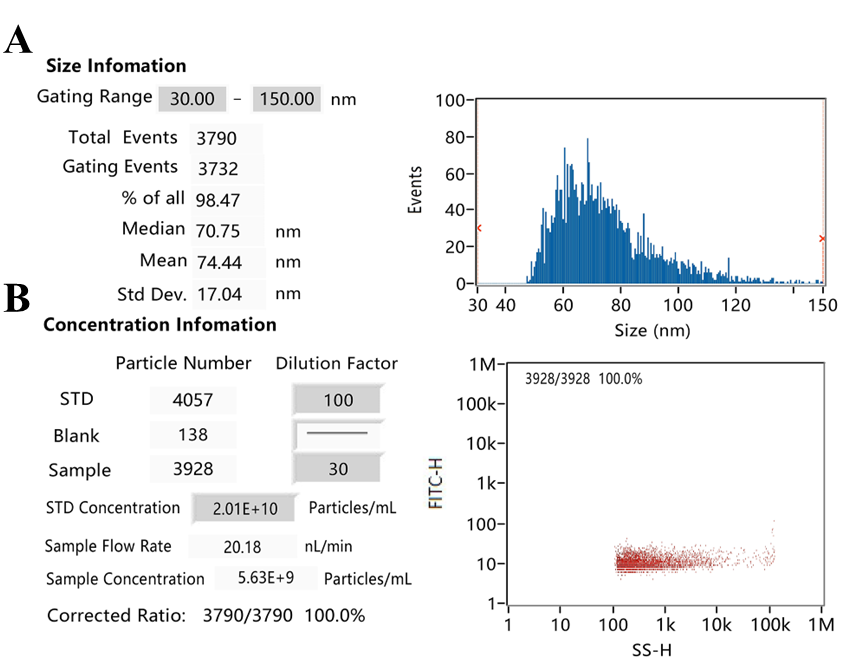


**Figure S1.** NTA analysis of purified exosomes from *H. pylori* infected GES-1 cells. (A) The particle diameter and (B) concentration of exosomes were determined with NTA.


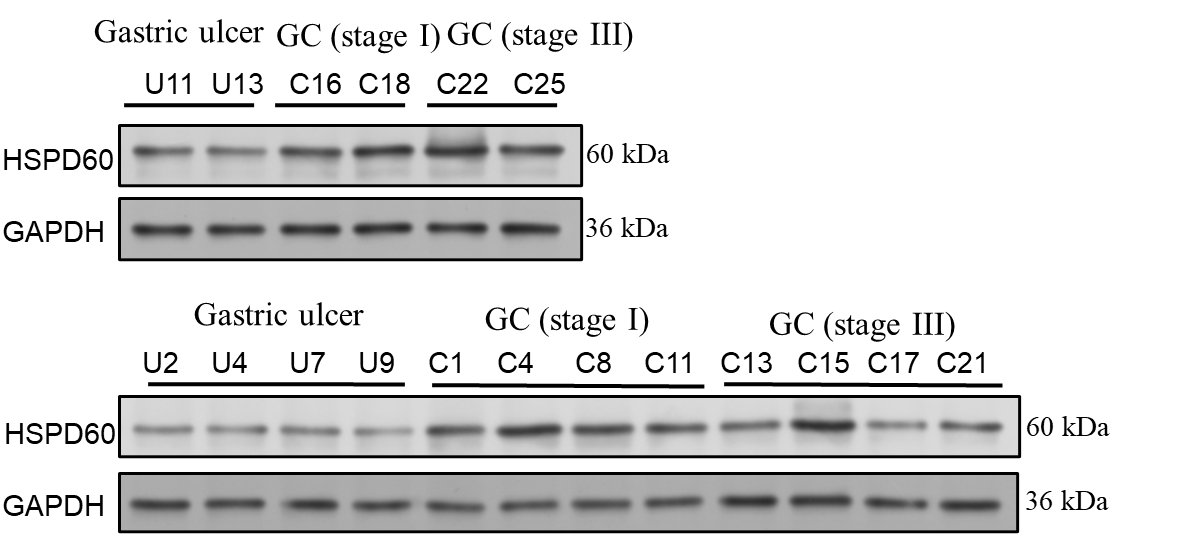


**Figure S2.** The infection of H. pylori regulates the expression of HSP60 in GC. The expression of HSP60 in gastric ulcer, and stage I and III GC mucosal tissues was determined by western blot.
